# Supplementary material for: Modeling glioblastoma heterogeneity as a dynamic network of cell states
Source: Mol Syst Biol. 2021 Sep 16;17(9):e10105. doi: 10.15252/msb.202010105 (PMC8444284; doi:10.15252/msb.202010105)
Supplement: Supplementary file 5 — Source Data for Figure 3 [file MSB-17-e10105-s001.zip › Figure3A_sourcedata/GSEA_3065/hallmarks_state3.GseaPreranked.1623416274744/HALLMARK_COAGULATION.html]

Details for gene set HALLMARK\_COAGULATION[GSEA]

|  || Dataset | state3 |
| Phenotype | NoPhenotypeAvailable |
| Upregulated in class | na\_pos |
| GeneSet | HALLMARK\_COAGULATION |
| Enrichment Score (ES) | 0.374865 |
| Normalized Enrichment Score (NES) | 1.3178923 |
| Nominal p-value | 0.0859375 |
| FDR q-value | 0.13831769 |
| FWER p-Value | 0.647 |
Table: GSEA Results Summary

  

Fig 1: Enrichment plot: HALLMARK\_COAGULATION      
 Profile of the Running ES Score & Positions of GeneSet Members on the Rank Ordered List

  

| PROBE | GENE SYMBOL | GENE\_TITLE | RANK IN GENE LIST | RANK METRIC SCORE | RUNNING ES | CORE ENRICHMENT || 1 | PLAT |  |  | 18 | 0.513 | 0.0562 | Yes |
| 2 | CD9 |  |  | 32 | 0.473 | 0.1083 | Yes |
| 3 | TIMP1 |  |  | 35 | 0.470 | 0.1613 | Yes |
| 4 | TFPI2 |  |  | 65 | 0.388 | 0.2020 | Yes |
| 5 | LRP1 |  |  | 152 | 0.309 | 0.2272 | Yes |
| 6 | LAMP2 |  |  | 154 | 0.309 | 0.2621 | Yes |
| 7 | ADAM9 |  |  | 229 | 0.272 | 0.2844 | Yes |
| 8 | SPARC |  |  | 361 | 0.228 | 0.2952 | Yes |
| 9 | CTSB |  |  | 392 | 0.219 | 0.3166 | Yes |
| 10 | PROS1 |  |  | 514 | 0.197 | 0.3250 | Yes |
| 11 | A2M |  |  | 585 | 0.188 | 0.3384 | Yes |
| 12 | FBN1 |  |  | 768 | 0.161 | 0.3357 | Yes |
| 13 | TIMP3 |  |  | 950 | 0.142 | 0.3311 | Yes |
| 14 | HTRA1 |  |  | 955 | 0.142 | 0.3467 | Yes |
| 15 | FN1 |  |  | 973 | 0.140 | 0.3607 | Yes |
| 16 | CLU |  |  | 988 | 0.139 | 0.3749 | Yes |
| 17 | CPQ |  |  | 1371 | 0.110 | 0.3434 | No |
| 18 | THBS1 |  |  | 1561 | 0.100 | 0.3331 | No |
| 19 | LGMN |  |  | 1637 | 0.095 | 0.3352 | No |
| 20 | BMP1 |  |  | 1722 | 0.090 | 0.3358 | No |
| 21 | CAPN5 |  |  | 1859 | 0.083 | 0.3296 | No |
| 22 | PRSS23 |  |  | 1890 | 0.081 | 0.3354 | No |
| 23 | MMP2 |  |  | 1898 | 0.081 | 0.3438 | No |
| 24 | C1R |  |  | 2407 | 0.060 | 0.2922 | No |
| 25 | CTSO |  |  | 2920 | 0.043 | 0.2383 | No |
| 26 | SERPINE1 |  |  | 3090 | 0.039 | 0.2232 | No |
| 27 | USP11 |  |  | 3829 | 0.019 | 0.1406 | No |
| 28 | CTSV |  |  | 4007 | 0.015 | 0.1219 | No |
| 29 | GSN |  |  | 4064 | 0.013 | 0.1170 | No |
| 30 | SH2B2 |  |  | 4334 | 0.006 | 0.0867 | No |
| 31 | MMP11 |  |  | 4837 | -0.007 | 0.0298 | No |
| 32 | SIRT2 |  |  | 4877 | -0.008 | 0.0262 | No |
| 33 | MMP15 |  |  | 4903 | -0.008 | 0.0243 | No |
| 34 | PLAU |  |  | 5363 | -0.020 | -0.0262 | No |
| 35 | LTA4H |  |  | 5492 | -0.024 | -0.0382 | No |
| 36 | FURIN |  |  | 5987 | -0.039 | -0.0906 | No |
| 37 | PREP |  |  | 6373 | -0.052 | -0.1290 | No |
| 38 | DUSP6 |  |  | 6502 | -0.056 | -0.1373 | No |
| 39 | RABIF |  |  | 6600 | -0.061 | -0.1416 | No |
| 40 | PEF1 |  |  | 6872 | -0.074 | -0.1643 | No |
| 41 | CSRP1 |  |  | 6916 | -0.077 | -0.1605 | No |
| 42 | FYN |  |  | 6924 | -0.077 | -0.1526 | No |
| 43 | ANXA1 |  |  | 7032 | -0.083 | -0.1554 | No |
| 44 | MSRB2 |  |  | 7268 | -0.098 | -0.1713 | No |
| 45 | DUSP14 |  |  | 7727 | -0.143 | -0.2078 | No |
| 46 | CAPN2 |  |  | 7878 | -0.162 | -0.2066 | No |
| 47 | WDR1 |  |  | 8058 | -0.195 | -0.2051 | No |
| 48 | ARF4 |  |  | 8128 | -0.211 | -0.1891 | No |
| 49 | GNG12 |  |  | 8323 | -0.264 | -0.1814 | No |
| 50 | CRIP2 |  |  | 8455 | -0.319 | -0.1603 | No |
| 51 | ISCU |  |  | 8531 | -0.352 | -0.1289 | No |
| 52 | GNB2 |  |  | 8612 | -0.415 | -0.0910 | No |
| 53 | S100A13 |  |  | 8652 | -0.457 | -0.0436 | No |
| 54 | RAC1 |  |  | 8673 | -0.484 | 0.0090 | No |
Table: GSEA details [plain text format]

  

Fig 2: HALLMARK\_COAGULATION: Random ES distribution      
 Gene set null distribution of ES for **HALLMARK\_COAGULATION**
